# Combined usage of Ho:YAG laser with monopolar resectoscope in the treatment of bladder stone and bladder outlet obstruction

**Published:** 2014

**Authors:** Jian Hui Wu, Kuo Yang, Qian Liu, Shi Qiang Yang, Yong Xu

**Affiliations:** 1 Jian Hui Wu, Department of Urology, Second Hospital of Tianjin Medical University, Tianjin Institute of Urology, No. 23 Pingjiang Road, Tianjin 300211, China.; 2 Kuo Yang, Department of Urology, Second Hospital of Tianjin Medical University, Tianjin Institute of Urology, No. 23 Pingjiang Road, Tianjin 300211, China.; 3 Qian Liu, Department of Urology, Tianjin First Center Hospital, No.24 Fukang Road, Nankai District, Tianjin 300192, China.; 4 Shi Qiang Yang, Department of Urology, Tianjin First Center Hospital, No.24 Fukang Road, Nankai District, Tianjin 300192, China.; 5 Yong Xu, Department of Urology, Second Hospital of Tianjin Medical University, Tianjin Institute of Urology, No. 23 Pingjiang Road, Tianjin 300211, China.

**Keywords:** Urinary bladder calculi, Prostatic hyperplasia, Urinary bladder neck obstruction, Lasers, Solid-state, Lithotripsy, Laser, Transurethral resection of prostate

## Abstract

***Objective:*** Bladder stones in elderly men are commonly associated with bladder outlet obstruction, and many different treatment modalities have been presented for both these conditions. To evaluate the effectiveness and safety of a novel method concerning spontaneous usage of both monopoplar transurethral resection of the prostate and Holmium Laser cystolithotripsy, we compared the transurethral use of resectoscope and cystoscope lithotripsy approaches retrospectively.

***Methods:*** Patients data of one hundred and nine male patients with benign prostatic hyperplasia (BPH) and bladder stone(s) were analyzed retrospectively. Two groups of patients were compared: Group I was treated with combination of transurethral holmium laser cystolithotripsy (HLC) and transurethral resection of the prostate (TURP) using the 24F resectoscope, and group II used 22F cystoscope and 24F resectoscope for treating both these conditions.

***Result:*** We reviewed the records of 109 patients undergoing transurethral cystolithotripsy with holmium laser and simultaneous TURP. The mean bladder stone size were 3.6±1.5 cm in Group-I and 3.7±1.1 cm (mean 3.8) in Group-II (p>0.05). The mean operation time of Group-I and Group-II was 49.0±22.5 minutes and 79.0±28.5 minutes, respectively (p<0.05). Stone fragments were removed completely and TURP procedures were done successfully in all of the patients. Mild hematuria was found more frequently in Group-II (22.2%), and four (7.4%) patients had urethral stricture in the same group during the late follow-up.

***Conclusion:*** Combination of transurethral laser cystolithotripsy and TURP using the same 24F resectooscope is an effective, safe and economical treatment for bladder stones in BPH patients. It is minimally invasive and involves and has lower complication rates and shorter hospital stay. However, this combined approach should be taken in the treatment of calculus within 4 or 5 centimeters.

## INTRODUCTION

Bladder stones in elderly men are often associated with bladder outlet obstruction (BOO) caused by benign prostatic enlargement (BPE), and treatment usually combines endoscopic removal of bladder stone and transurethral resection of the prostate (TURP) for managing the BOO. Although in many cases the stone burden are not large than 4cm, proper approach that can treat BPH and bladder stones simultaneously, may be even more challenging. Both the duration of TURP and the coexisting illnesses of these patients demand efficient lithotripsy within a limited time.

Transurethral cystolithotripsy is the most prevalent uesd approach to manage cystolithiasis. This approach permits the use of diverse tools for stone fragmentation, including mechanical lithotripters, electrohydraulic lithotripsy, pneumatic/ ultrasonic lithotripter and laser energy.^1^ The widely accepted clinical lasers for lithotripsy are the holmium: YAG (Ho:YAG). It is an ideal intracorporeal lithotriptor for all types of stones as well as large and hard bladder calculi.^2^ Conventionally, transurethral cystolithotripsy with holmium laser is performed by cystoscope or the 26 F resectoscope with a laser bridge. Although Ho:YAG laser lithotripsy can effectively fragment bladder stones, especially for the large bladder stones (>2cm), it is time consuming to evacuate such stone fragments through the cystoscope sheath. Therefore, Aron et al.^3^ introduced percutaneous suprapubic cystolithotripsy as an alternative option for very large stones.

Subsequently, Kamat et al.^4^ reported that the suprapubic route were easier and faster retrieval of large stone fragments. However, it is more invasive than the transurethral way. Meanwhile, diverse modifications were developed on the technique of transurethral cystolithotripsy. These modalities are described to facilitate evacuation of larger stone fragments, including the use of a resectoscope with a stone basket^5^, the Urovac bladder evacuator attaching to the standard 30F Amplatz working sheath, the use of an Amplatz sheath through the male urethra and using a laparoscopic entrapment sac.^5^^,^^6^ All these approaches typically use resectoscope or nephroscope larger than 26F, and the urethra dilatation is performed to 30F. In some cases, urethrotomy should be considered to widen the distal urethra.

All the literatures reported the bladder stones can be effectively treated, but problem is the lack of follow-up study of these approaches and the postoperative urethral strictures is not commonly noted. In fact, use of larger size nephroscope or resectoscope sheath may cause urethral stricture in long term outcome.^1^ Because of these issues, we sought to develop a simple and effective method for removal of bladder calculi with simultaneous TURP.

## METHODS

A total of 109 men with BOO and associated bladder stones were treated at our institution between May 2005 and December 2013. The diagnosis of BOO was based on the presence of significant lower urinary tract syndrome (LUTS) and obstructed uroflowmetry rates. All patients were evaluated by a medical history including the international prostate symptom score (IPSS), quality of life (QoL) score and physical examination, including a digital rectal examination (DRE), prostate specific antigen (PSA) assay, urine analysis, urine culture and ultrasonography. Once a bladder stone was identified, CT or urethrocystoscopy was performed in all patients to determine the size and number of calculi. MR urography (MRU) or intravenous urography (IVU) was used selectively to evaluate the upper urinary tract in patients. Patients with urethral stricture, a preoperative diagnosis of prostate cancer, neurogenic voiding dysfunction, stone greater than 5cm and those associated upper tract stones were excluded from the study. Under epidural or general anesthesia (due to preoperative anticoagulant therapy), the patients were placed in the lithotomy position.

**Fig.1:**
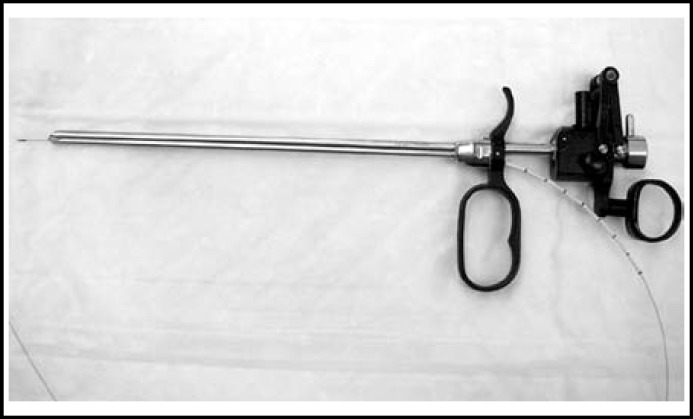
The 550μm laser fibre is inserted into the working channel of the monopolar resectoscope through the ureteral catheter

Patients were stratified retrospectively into two groups according to the method of stone fragmentation. Group-I included 55 patients undergoing transurethral cystolithotripsy with holmium laser and simultaneous TURP using the 24F resectoscope, and Group-II consisted of 54 patients who used 22F cystoscope and 24F resectoscope for treating both these conditions. In Group-I the procedure was started by transurethral cystolithotripsy with holmium laser. We cut a 5Fr ureteral catheter for about 10cm length. This was then inserted into the cutting loop channel of the 24F monopolar resectoscope (Richard Wolf, Knittlingen, Germany). A 550-micrometer laser fiber was then inserted into the working cavity through the ureteral catheter lumen. (Fig.1). Bladder Stones were fragmentated using 100W laser generators (VersaPulse PowerSuite 100W, LUMENIS Surgical, Santa Clara, CA, America) with settings of 0.5 to 1.5 J and 10 to 25 Hz. Once the stone is fragmented, we use the Urovac bladder evacuator (Boston Scientific, Natick, MA, American) attach to the resectoscope sheath. The bladder was first partially filled through the resectoscope sheath. Then the fragments are flushed out from the sheath. Subsequently, the working element was removed and cutting loop inserted into the work channel, while the resectoscope sheath was left in the urethral for the next step. This was followed by TURP with the same resectoscope. In Group-II , the procedures were applied with transurethral use of 22F cystoscope (Karl Storz, Tuttlingen, Germany). The same holmium laser device was used in all patients for stone fragmentation. This procedure often necessitates a number of transurethral entries to pull the fragments out. Subsequently, TURP was carried out. At the end of operation, a 22F Foley catheter was left in the urethral and bladder.

Statistical comparison of both groups was performed by Mann-Whitney u-test. Statistical analyses were performed using SPSS (SPSS, Chicago, IL, USA) software version 19. A p-value less than 0.05 was considered statistically significant.

## RESULT

Each treatment group displayed similar demographic and baseline characteristics (Table-I). Stone fragments were removed completely and TURP procedures were done successfully in all the patients. Mean patient age at the time of diagnosis was 52.0±11.8 years in Group-I and 53.6±10.4 years in Group-II (p=0.729). The bladder stone size were 3.6±1.5 cm (mean 3.9) in Group-I and 3.7±1.1 cm (mean 3.8) in Group-II . The mean operation time was 49.0±22.5 minutes in Group-I and 79.0±28.5 minutes in Group-II. The difference between the mean operation time of two groups was statistically significant (p<0.01). The average weight of prostate tissue removed in Group-I was 34.2±8.5 g and that in Group-II was 35.2±4.8 g (p=0.920). The mean resection time of BPH was 42.6 ±18.5 minutes in Group-I and 43.2±19.2 minutes in group 2(p=0.624). The lithotripsy and evacuation time was 25.8±15.6 minutes in Group-I and 36.4±19.3 minutes in Group-II , respectively (p<0.01). Patients in Group-II had significantly higher duration of catherization and hospitalization. And the incidence of post-operative discomfort were occur more frequently in Group-II, including burning on catheterization, bladder pain, dysuria or hematuria after catheter removal.

No intraoperative major complications such as bladder perforation or significant mucosal lesions were observed in any of the patients. However, mild hematuria was found more frequently in cystoscopy cystolithotomy (12.7% in Group-I , 22.2% in Group-II). No patients required a blood transfusion. Patients who had blood cultures with positive results were given antibiotics for 10-14 days postoperatively. Peri-operative complications are also listed in Table-I.

The patients were followed for at least one year (mean 1.4 year). The LUTS, peak urinary flow rate (Qmax) and PVR was significantly improved at 1 to 3 months after surgery and these immediate improvements were significant for each variable (P<0.001, data not shown). Early complications related to the procedure were observed in 39 patients and consisted of postoperative hematuria in nineteen (seven in Group-I and twelve in Group-II ) without significant change in the hematocrit, urinary tract infection in fourteen (three in Group-I and eleven in Group-II ) and clot retention in two (one in each group). During the late follow-up, four patients had urethral stricture and showed decreased flow rates. All of these four patients were in Group-II, who had no urethral stricture disease preoperatively. The places of the stricture were all the same as posterior urethral strictures. An Otis urethrotome was used and the urethra calibrated to 26 F.

In Group-I only one transurethral entry was made throughout the entire procedure via resectoscope in all patients. In Group-II, two or more transurethral entries were needed in the 54 patients (Table-I).

## DISCUSSION

The European Urology Association guideline considers bladder stone as a complication of BPH and strongly recommends surgical management of BOO in the presence of bladder stones.^7^ There are varieties of options available for management of both these conditions. However, surgery usually combines endoscopic removal of bladder calculi and TURP for managing the BOO.^8^ Combined procedures for treating BPH-related BOO and bladder calculi is a practicle solution, as it appears to be favourable for both surgeon and patient, requiring only one anaesthetic and a short hospital stay.^9^^,^^10^

Several innovations in TURP technique have been described in the past few decades, including bipolar techniques and transurethral enucleation technique, but, monopolar TURP remains the gold standard.^11^ However, there are varieties of options available for management of bladder stone and there is no agreement about the preferred method of treating this condition in patients with associated BOO.^1^ Various methods attempted for bladder stones include shockwave lithotripsy (SWL), transurethral cystolithotripsy, percutaneous cystolithotripsy or open surgery. Each method has its advantages and disadvantages. The choice of surgical approach and method of stone fragmentation is based on availability of equipment, surgical experience, patient characteristics (age, comorbidity, and anatomy), and stone parameters (size and composition).^12^

**Table-I:** The patients’ demographics and operative characteristics, and the complications during and after surgery

	***Group 1*** ***(n=55)*** ***Mean or n(%)***	***Group 2*** ***(n=54)*** ***Mean or n(%)***	***p-Value***
Age (years)	52.0±11.8	53.6±10.4	0.729
*Preoperative:*			
IPSS score	19.6 ± 6.4	19.5±7.6	0.814
QoL score	4.3 ±1.2	4.4±1.4	0.829
Qmax, mL/s	6.9±4.3	6.3±4.1	0.761
PVR, mL	193±26.39	192.6 ± 27.19	0.897
Bladder stone size (cm)	3.6±1.5	3.7±1.1	0.618
Prostate weight (g)	62.0 ± 7.1	64.0 ± 5.9	0.952
Operation time (minutes)	49.0±22.5	79.0±28.5	0.021
Time of Lithotripsy and evacuation (minutes)	25.8±15.6	36.4±19.3	0.040
Time of TURP (minutes)	42.6±18.5	43.2±19.2	0.624
weight of prostate tissue removed (g)	34.2±8.5	35.2±4.8	0.920
Transurethral access (number of access)	55	172	0.000
Postoperative duration of catherization (day)	4.5±1.5	7.±2.2	0.042
Duration of hospitalization (day)	9.0±4.5	16±5.5	0.033
*Complications:*			
Intraoperative mild hematuria	7(12.7%)	12(22.2%)	
Urinary tract infection	3(5.4%)	11(20%)	
Clot retention	1(1.8)	1(1.9%)	
Posterior urethral stricture	0(0%)	4(7.4%)	
Total	11(20%)	28(51.8%)	

The transurethral approach for vesical calculi treatment using a natural orifice for access is incisionless and always attractive. Currently, it is probably the most common way to manage bladder stone. The relevant issues in this approach include choice of lithotripsy device and the choice of instrument to gain access to bladder.

Holmium laser cystolithotripsy (HLC) and pneumatic/ultrasonic lithotripsy are the most commonly employed for managing urinary stone disease in endourology. Many studies had validated the safety and efficacy of combining TURP with pneumatic lithotripsy. However, this device had limitations in treating large and hard stone.^10^ Ener et al. and Kingo et al.^13^^,^^14^ used lithoclast master that combines pneumatic and ultrasonic lithotripsy and also give advantage of aspiration thereby improving vision during surgery. Holmium laser is now considered an intracorporeal lithotripsy device of choice. Shah et al.^9^ combined holmium laser enucleation of the prostate (HoLEP) with holmium laser lithotripsy in treating 32 patients with bladder stones secondary to BPH. They found the combination to be safe and effective, and concluded that stones of any size and composition and prostates of practically any size can be treated endoscopically using holmium laser once the technique is mastered. While, to our experiences and according to this study, transurethral procedure cannot satisfactorily manage stones over five centimeters within a limited time.

Generally cystoscope is used for transurethral lithotripsy. The major drawback of cystoscope is its smaller lumen that makes evacuation of stone fragments difficult and time consuming. In the Group-II of our study, it was difficult to remove the larger stone fragments out, and necessitates pulling the cystoscope out together with the stone at its tip to the urethral meatus. Fragmented stones may damage the urethra during this process, especially when they escape from the grasping forceps, because at this time surgeon would need to make an additional maneuver to grasp or fragment the stone in the urethra. For that reason, the surgeon has to fragment the bladder stone as much as possible while using the cystoscope to avoid forceps use and to evacuate small fragments by irrigation and aspiration. However, worsening visual quality during the procedure parallel to the amount of stone fragmentation. The narrow cystoscope sheath and its small caliber scope are the main disadvantages of treatment for larger vesical calculi. To overcome this problem, using larger sheaths and endoscopes in bladder is preferred treatment modality, which provides a better irrigation flow and vision, and allows the use of larger forceps to gather large fragmentations. Chtourou et al.^10^ have presented transurethral penetration with a 26F nephroscope as a novel minimally invasive technique in the management of large bladder stones. They reported combining management of bladder calculi and BPH by ballistic lithotripsy (BL) and TURP was effective and safe. Okeke et al.^15^ successfully placed 30 F Amplatz sheath in male urethral for direct access to bladder in five patients with average stone burden of 6.7 cm. They used 26 F nephroscope through the Amplatz sheath for stone fragmentation. Three out of these five patients had chronic bladder outlet obstruction secondary to BPH. All patients were stone free and at average follow-up of 9.4 months no patients developed urethral stricture. Ener et al.^14^ compared transurethral nephroscope vs. cystoscope for transurethral management of stone >2 cm in largest diameter. Nephroscope was found to yield faster stone treatment by allowing use of larger forceps and facilitating removal of large fragments through 24F sheath. Shah et al.^9^ reported the procedure of HoLEP combined with HLC. Before the procedure an Otis urethrotome was used and the urethra was calibrated to 30 F. During combined HoLEP with transurethral HLC, only one transurethral access was made throughout the entire procedure via 26F resectoscope in all patients, but the holmium laser enucleation of the prostate is associated with a steep learning curve and it is also the most expensive modality for such patients. As in all the endoscopic interventions, a potential complication of transurethral method in the treatment of bladder stones is the iatrogenic injury of the urethral lumen. This complication may develop depending on the device used and the size of the stone. In addition, reinserting the cystoscope also has an additional risk for urethral damage. Authors hypothesized that the higher number of transurethral entries needed during cystoscope use may increase possibility of urethral trauma causing stricture in long term.^16^ However use of larger size nephroscope sheath may have the same effect. Kingo et al.^13^ confirmed the safety of transurethral use of 24 F nephroscope. In our study, the 24F resectoscope also showed realizable and effective in this combined procedure. The time of operation and hospitalization of Group-I were significantly shorter than Group-II and there was no postoperative urethral stricture in follow-up result of Group-I.

When it was compared with those discussed above modalities, we found that using the 24F resectoscope was associated with a number of benefits. First, the 24F momopolar resectoscope is almost a “one size fits all” instrument. It does not require any other equipment, such as a special laser bridge, a cystoscope with catherter element. During the whole procedure, the resectoscope sheath need not be changed, decreasing the urethral entries and the risk of damage. Secondly, the 24F momopolar resectoscope offered a larger vision, the rinsing water system of the resectoscope maintains a clearer view of the operating zone. As a result, higher laser settings than those common in clinical practice can be used. These also may shorten operation time.^17^ Finally, the attachment point of the 5Fr ureteral catherter fits perfectly into the cutting loop channel, such that a stablized laser channel is formed. The laser fiber is preferably protected and can avoid being broken. Once the stone is fragmented, the Urovac evacuator was attached to the rescetoscope sheath, permitting the rapid evacuation of stone material from the bladder, much as when the Ellik device is used to evacuate prostate chips during a transurethral resectoscope of prostate procedure. Therefore, more efficient fragments removal will likely minimize the likelihood of residual stone material and reduce the operative time.

However, the method used in this study was not evaluated for stones of mean size over 5 cm. The pcutaneous suprapubic cystolithotripsy is preferable in patients with large vesical calculi. There are two studies in literature that compared pneumatic lithotripsy by suprapubic vs. transurethral route along with TURP in managing large bladder stones and associated BPH.^3^^,^^4^ Both these studies found percutaneous cystolithotripsy to be rapid, safe and effective. In addition, Richter et al.^18^ combined open cystolithotomy with TURP in treating patients with very large bladder or numerous bladder stones and enlarged prostate. The author concluded that it is a quick procedure and should still be a procedure of choice in treating patients with very large or numerous bladder stones. There was bias in this study criteria due to the retrospective nature of the study. Approach in each case should be individualized based on the patient’s clinical criteria and his choice, availability of various endoscopy modalities, surgical expertise and experiences.^1^

## CONCLUSION

The transurethral holmium laser cystolithotripsy with 24F resectoscope combined with TURP is considered as an effective, safe and economical modality for managing the bladder calculi with BPH.

## Author’s contributions:

Jian Hui Wu and Qian Liu invented the combined operating technology.

Jian Hui Wu, Qian Liu and Yong Xu designed the study, performed the operation and prepared the manuscript.

Jian Hui Wu and Kuo Yang contributed to the writing and to the critical reading of the paper.

Shi Qiang Yang and Kuo Yang were involved in patient collection and clinical data interpretation.

Kuo Yang performed the statistical analysis. All authors read and approved the final manuscript.

## Notes

***Disclosure Statement: ***The authors declare that they have no competing interests.
